# Social jetlag affects jump skills in sub-elite volleyball players

**DOI:** 10.3389/fspor.2024.1443804

**Published:** 2024-07-30

**Authors:** Andrea Ciorciari, Lucia Castelli, Letizia Galasso, Antonino Mulè, Fabio Esposito, Eliana Roveda, Angela Montaruli

**Affiliations:** ^1^ Department of Biomedical Sciences for Health, University of Milan, Milan, Italy; ^2^ Faculty of Education, Free University of Bozen-Bolzano, Brixen-Bressanone, Italy; ^3^ Division of Exercise Physiology, School of Medicine, West Virginia University, Morgantown, WV, United States

**Keywords:** social jetlag, performance, volleyball, circadian rhythm, sport

## Abstract

Social jetlag (SJL), resulting from misalignment between biological rhythms and social schedules, has emerged as a prevalent phenomenon in modern society, particularly among young athletes. However, the effect of SJL on performance is poorly studied. Jump and dynamic balance are two key skills in volleyball, as the first allows the player to perform better both during the offense and defense phase, and the second is fundamental in landing and in injury prevention. Therefore, our aim was to investigate the effect of SJL on jump skill performance and balance in female volleyball players. Thirty female volleyball players (mean age: 17.3 ± 0.88 years) participated in the study. SJL was assessed using the Munich ChronoType Questionnaire (MCTQ), integrated with Jankowsky's sleep-corrected formula. Jump skill performance was evaluated using a standardized jump test, the Vertec Jump Test, while balance was assessed with the Y Balance Test. The tests were performed at 09:00 a.m. and at 06:00 p.m. The results revealed that players with greater SJL exhibited decreased jump performance, characterized by lower vertical jump height (*p* = 0.02). Furthermore, players with lower SJL showed the typical difference between morning and afternoon performance (*p* = 0.001), demonstrating their synchronization between biological rhythms and social commitments, while no statistically significant difference between the two sessions was shown in players with higher SJL. Regarding balance, no significant association with SJL was found, but the morning session yielded lower results than the afternoon one (*p* = 0.01). These findings highlight the detrimental impact of SJL on jump skill performance, underscoring the importance of optimizing sleep-wake schedules and circadian alignment to enhance athletic performance. Future research should explore targeted interventions, such as sleep hygiene education, to minimize social jetlag and promote optimal performance in adolescent athletes.

## 1 Introduction

Social Jetlag (SJL) is the misalignment between biological and social clocks (1). It is mathematically calculated by comparing the mid-sleep phase of workdays and free days, and recently this formula was updated with a sleep-correction, including the sleep debt accumulated during the workdays (2). A person with a high SJL will manifest symptoms similar to sleep debt and jetlag, which are both factors which seem to influence athletic performance (3). Athletes often experience sleep problems such as insufficient sleep and insomnia, leading to excessive sleepiness, daytime dysfunction, and performance problems (4). They tend to take longer to fall asleep, spend more time awake, have reduced sleep efficiency, and experience greater sleep fragmentation (5). This condition can disrupt their biological and social rhythms, causing a circadian misalignment and SJL. Additionally, athletes face other chronobiological factors that contribute further to performance, such as the chronotype (CT) (6), a genetically determined predisposition influencing an individual's preference for activity times. Morning-types (M-types) prefer early activity and sleep times, Evening-types (E-types) prefer later activity and sleep times, and Neither-types (N-types) fall in between (7). The effects of sleep on performance and injury prevention are widely studied, as it seems that chronic poor sleep can decrease athletic performance (8, 9) and increase injury risk (10). Few studies investigated the effects of SJL on performance and injury risks, but it seems that it could impair balance (11) and raise injury risk (12). However, SJL is poorly studied in sports, although it may reasonably affect performance and injury risk by acting on the same physiological and psychological mechanisms as sleep.

Volleyball is a team sport involving short-duration maximal and explosive movements, many of which include vertical jumps during continuous offensive and defensive actions (13). Numerous studies highlight that good sleep quality is essential for better physical and emotional recovery in athletes. Andrade and colleagues (14) investigated the impact of sleep on volleyball performance and found that players with a poorer perception of their sleep quality experienced greater pre-match disturbance compared to those with good sleep quality. Insufficient sleep has also been linked to increased fatigue and tension during competitions. Scott and colleagues (15) reported an inverse relationship between sleep deprivation-induced fatigue and reaction time, which is a critical skill in volleyball. Moreover, volleyball is a sport with a high risk of injuries, and sleep alterations can generally reduce postural and dynamic motor control (16, 17), increase lapses in attention and mental fatigue (18, 19), lead to impulsivity and risk-taking behaviors (20–22) and decrease several indices of sports performance, creating a condition that increases injury risk (23–25). Therefore, promoting a good sleep schedule and regular circadian rhythms seem to be a key factor in optimizing performance and injury risk prevention in volleyball. The timing of athletic performance is another factor influencing outcomes, and CT seems to play an important role (26–28). A recent study (13) tested volleyball players at 9 a.m. and 7 p.m. on short-duration maximal performances. Results indicated better neuromuscular performance in the evening session in terms of spike test without a jump, flexibility, dynamic balance, and agility, while there was no significant difference in vertical jump ability and isometric strength.

Currently, there is significant scientific evidence on the effects of sleep alterations on sports performance and specific motor skills but none on the effect of SJL. Moreover, volleyball players are a population poorly studied both in terms of sleep and SJL. Therefore, this study aims to investigate the effect of SJL on jump skill performance, which is a fundamental ability in volleyball, and balance, which is a key factor in injury prevention, in female volleyball players. Given the detrimental impact of SJL on circadian rhythms, we hypothesize that individuals with higher SJL will exhibit lower performance in both balance and vertical jump. Additionally, we anticipate that individuals with low SJL, who are more synchronized with societal schedules, will demonstrate the typical time-of-day effect, showing a difference in performance between morning and afternoon. Conversely, we do not expect this time-of-day performance variation in individuals with high SJL.

## 2 Materials and methods

### 2.1 Participants

For this study, a total of 30 female volleyball players (age = 17.3 ± 0.88 years) from two different sports clubs in the province of Milan were recruited. All participants were highly trained sub-elite players competing in national competitions (29), with a training schedule that included four weekly training sessions and a weekend match. Individuals with medical conditions that could affect jump performance, balance, sleep or circadian rhythms were excluded from this study. Informed consent was obtained from all participants prior to their involvement, and they were allowed to withdraw from the study at any time.

### 2.2 Assessment of social jetlag, sleep and chronotype

After a brief anamnesis, participants were asked to complete the Munich Chronotype Questionnaire (MCTQ) (30). The MCTQ is a validated self-report questionnaire designed to estimate an individual's circadian rhythm and sleep habits, including sleep timing on workdays and free days, sleep duration, CT and SJL. Then, SJL was corrected with Jankowski's sleep-corrected formula (2):

SJL = |sleep onset on free days| - |sleep onset on workday|

### 2.3 Assessment of jumping skill and balance

The physical tests were scheduled during the same week of the anamnesis and questionnaire completion. They were performed twice in two different sessions, at 09:00 a.m. and 06:00 p.m, and were always monitored by the same operator. All tests were preceded by a warm-up designed to increase body temperature and participants' attentiveness, including small jumps, running and mobility exercises to prepare the joints involved in jumping. Additionally, participants were given proper familiarization with the tests. Jumping skill was evaluated using the Vertec Jump Test (VJT) (31), a widely used method for assessing vertical jump performance. Participants performed a countermovement jump and touched the highest possible vane on the Vertec apparatus (Conquest OS 1032B, Italy). The height of the jump was recorded in centimetres. Participants were allowed to attempt the jump three times, with at least 3 min of rest in between each attempt. In another session, balance was assessed using the Y Balance Test (YBT) (32), a reliable and valid measure of dynamic balance and neuromuscular control. Participants performed the Y Balance Test according to standardized protocols, reaching as far as possible along three directions (anterior, posteromedial, and posterolateral) while maintaining a single-leg stance on a grid. Then, the Absolute Reach Distance was extrapolated from the mean of the three measures.

### 2.4 Statistical analysis

The statistical analysis was performed according to the main aim of the study, i.e., investigating the effects of SJL on performance in the morning and the afternoon. Descriptive statistics (mean ± S.E.) were calculated for all variables of interest. The normality of the distribution of the data was assessed by Shapiro-Wilk Test. The Levene's Test was used to evaluate the homogeneity of variance of the data.

The sample size was calculated using GPower software (version 3.1.9.7). Considering the repeated measures ANOVA analysis, an effect size of 0.04 with a power of 0.95, an α error of 0.05, a confidence interval of 95%, two groups and two timepoints measurements, the software suggested a sample size of 24 participants. To account for potential dropouts and incomplete data for some participants, we recruited 30 subjects, surpassing the sample sizes suggested by the software.

Pearson correlation analysis was performed to examine the relationship between social jetlag, jump skill, and balance. Participants were categorized according to their SJL in two groups: all those who had a SJL above one hour (33) were considered people with high SJL (HSJL), while all the others were considered people with low SJL (LSJL). Repeated Measures ANOVA was used to compare HSJL and LSJL in the two sessions. Also, comparisons and correlations regarding sleep and chronotype were done Statistical significance was set at *p* < 0.05. The analysis was performed using SPSS Statistics version 29 (IBM SPSS Statistics for Windows, Armonk, NY, USA: IBM Corp).

### 2.5 Ethical approval

The study was carried out in accordance with the tenets of the 1964 Declaration of Helsinki and approved by the Ethical Review Board of the University of Milan in the September 2022 session, number 82/22.

## 3 Results

The total sample (*n* = 30; age = 17.3 ± 0.88 years; BMI = 25.01 ± 0.51 kg/m2; weight = 62.33 ± 1.6 kg; height = 1.7 ± 0.01 m) was categorized according to SJL (HSJL = 18; LSJL = 12). As previously said, the cut-off used to distinguish the two groups was one hour. Regarding the chronotype, 10 subjects were categorized as E-types, 3 as M-types and 17 as N-types. Table 1 displays the descriptive statistics of the whole sample and the two groups, as well as all the results of the tests in the two sessions.

**Table 1:** Descriptive statistics of the sample.

Whole sample (*n* = 30)			LSJL (*n* = 18)		HSJL (*n* = 12)	
	Mean	S.E.	Mean	S.E.	Mean	S.E.
Age (years)	17.33	0.88	18.83	1.25	15.08	0.84
BMI (kg/m2)	25.02	0.51	24.49	0.61	25.79	0.86
Weight (kg)	62.33	1.6	63.17	1.83	61.08	2.98
Height (m)	1.70	0.01	1.72	0.01	1.66	0.02
Social jetlag (hh:mm)	01:04	0:06	00:41	00:04	01:38	00:06
Vertec jump test a.m. (m)	2.60^§^	0.02	2.63˚	0.02	2.56	0.03
Vertec jump test p.m. (m)	2.64^§^	0.02	2.70˚^#^	0.02	2.58^#^	0.03
Vertec jump test mean (m)	2.62	0.02	2.67^×^	0.02	2.57^×^	0.03
Y balance test a.m. (cm)	95.25*	3.16	96.6	3.85	93.22	5.53
Y balance test p.m. (cm)	102.16*	2.78	102.93	3.06	101.5	3.3
Y balance test mean (cm)	98.56	2.45	99.76	3.09	97.36	3.79

§ *p* = 0.002.

*p = 0.01.

˚*p* < 0.001.

^#^ *p* = 0.007.

^×^*p* = 0.02.

**Figure 1:**
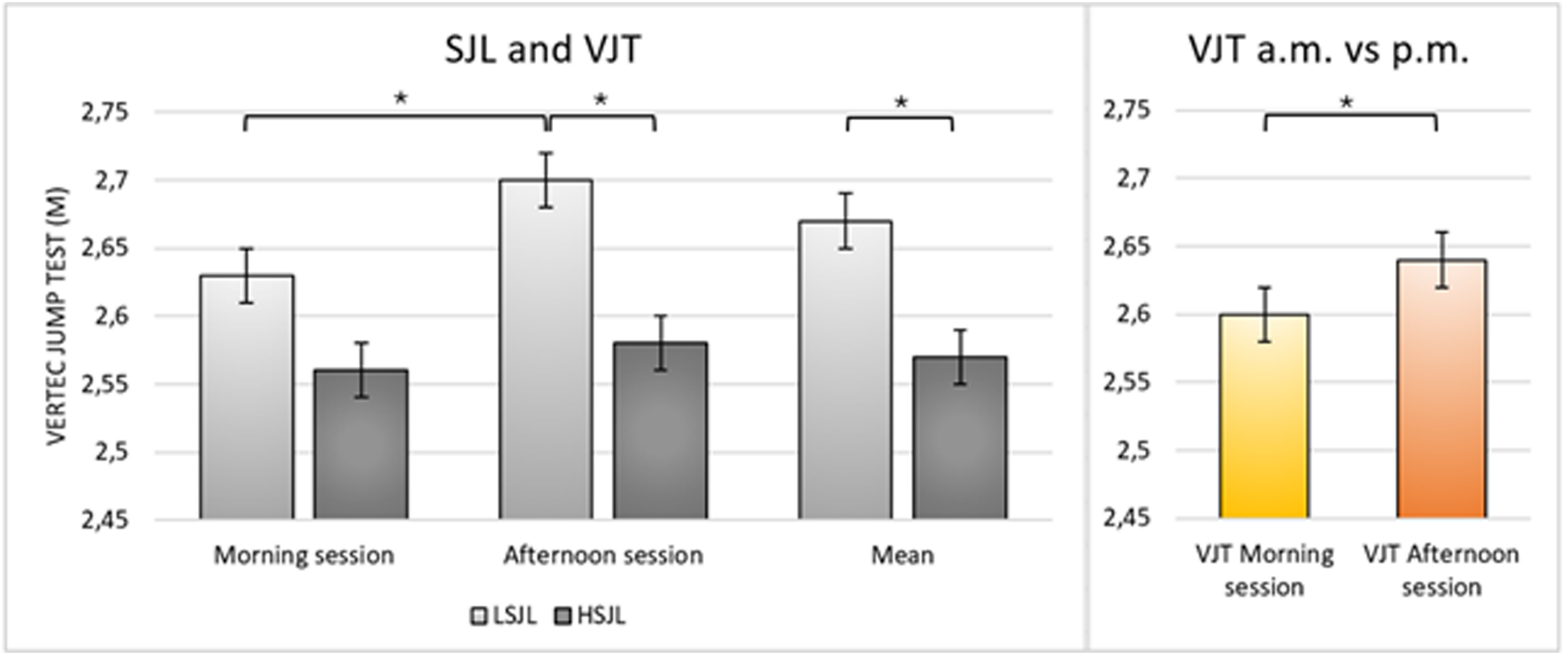
Comparisons between LSJL and HSJL in the two sessions of the VJT. In the comparison within group, LSJL performed better in the afternoon, while no difference was shown in HSJL. In the comparison between group, in the afternoon session LSJL performed better than HSJL. Considering the mean of the two sessions, LSJL performed better than HSJL. Considering the whole sample, the afternoon session showed greater results.

In the within group comparisons, when including all participants and comparing the results of the VJT, the afternoon session yielded higher results than the morning session (df = 1; *η*2 = 0.29; F = 11.68; *p* = 0.002). Moreover, LSJL performed better in the afternoon session than in the morning session (df = 1; *η*2 = 0.4; F = 18.89; *p* < 0.001), while no statistically significant differences emerged in the HSJL group (Figure 1).

In the between group comparisons, considering the average of the results of the two sessions and comparing HSJL and LSJL, the former performed worse than the latter in the VJT (df = 1; *η*2 = 0.18; F = 5.95; *p* = 0.02)(Figure 1). Moreover, in the afternoon session, HSJL performed worse than LSJL (df = 1; *η*2 = 0.23; F = 8.31; *p* = 0.007), while in the morning session, no statistically significant differences emerged between the two groups, (Figure 1).

**Figure 2:**
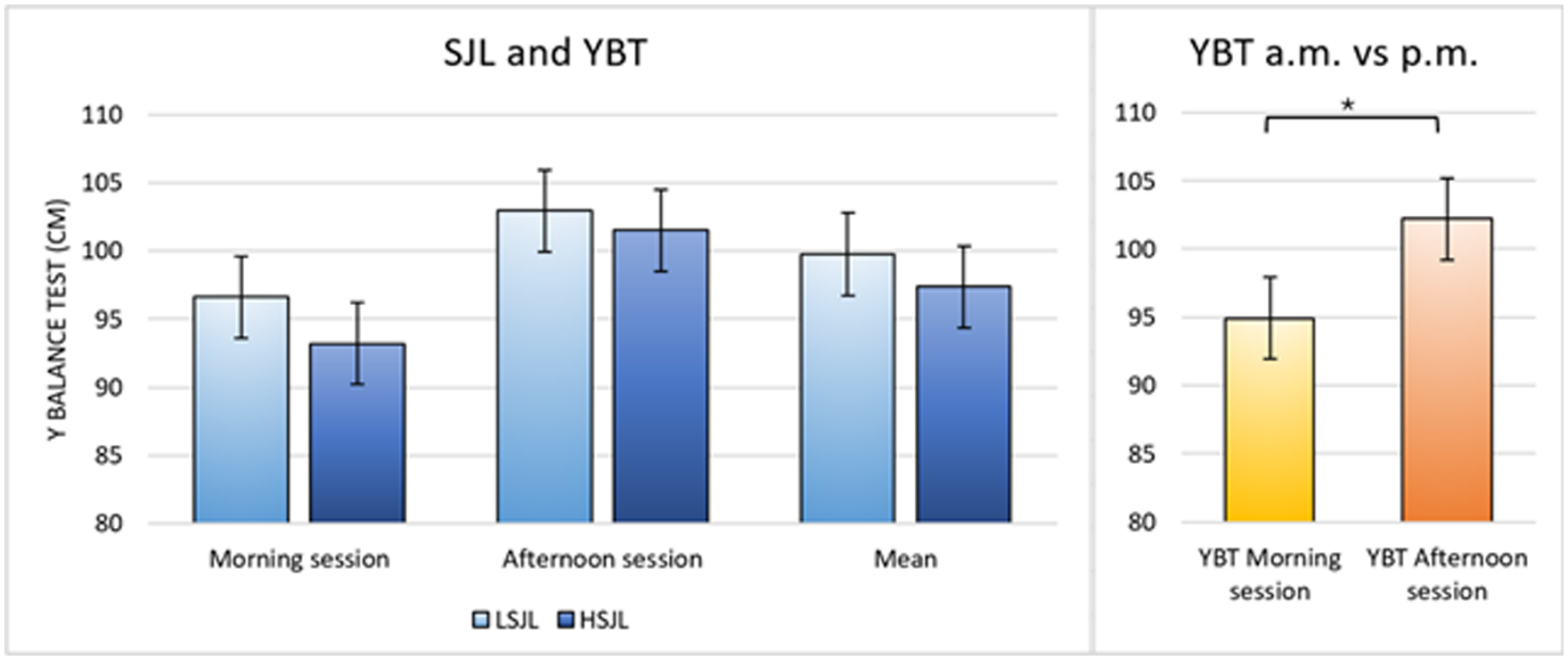
Comparisons between LSJL and HSJL in the YBT. No significant differences were found either between or within group. A statistically significant difference was observed when comparing the whole sample in the two sessions, with better results in the afternoon.

Comparing the two groups in the two sessions in the YBT, a statistically significant difference emerged in the comparison between morning and afternoon sessions including the whole sample (df = 1; *η*2 = 0.19; F = 6.63; *p* = 0.01), while comparing HSJL and LSJL no differences were shown (Figure 2).

No significance was found in the correlations and comparisons related to sleep, jump skill, balance and chronotype.

## 4 Discussion

The aim of this study was to evaluate the effects of SJL on balance and jumping skills in volleyball players in the morning and in late afternoon. To better characterize the sample and to strengthen the analysis, CT and sleep were also taken into account.

Our results indicate that both the VJT and YBT tests showed better performance in the afternoon session compared to the morning session, regardless of social jetlag. This outcome was expected, as many performance determinants, such as body temperature and hormonal secretion, follow a circadian rhythm, defining the peak of performance in the afternoon, according to the *time-of-day* effect (34). However, the novel findings emerged from the comparisons between LSJL and HSJL and within the groups across the two sessions. Specifically, in the overall mean of the two VJT sessions, HSJL performed worse than LSJL, indicating a significant impact of SJL on jumping skills. Moreover, the afternoon session for VJT highlighted a difference between the two groups, with LSJL performing better. Few studies in the literature have investigated the effect of SJL on performance (11, 12). The effects of SJL on physiology are linked to factors common to sleep restriction/deprivation and shift work (1). Consequently, we expected these results, as sleep impairments can decrease performance by affecting thermoregulation and hormonal responses (35). However, more detailed analyses of biochemical markers and hormone concentrations are needed to elucidate this relationship.

Although the difference might seem small, as LSJL had a mean jump of 2.70 cm while HSJL of 2.58, it must be considered that a difference of 12 cm can heavily affect both offensive and defensive phases in volleyball, potentially impacting the final outcome of a volleyball match. In contrast, the effect of SJL seemed lower in the morning, as no statistically significant difference between the two groups was found in either test. These results are consistent with previous ones on the effect of sleep on performance, since it seems that the greatest effect of sleep impairment is observed in the afternoon (26, 34), probably because of the altered cortisol secretion (36).

Another important finding is related to the effect of time of day within the two groups. LSJL showed the typical difference between morning and afternoon performance in VJT, demonstrating synchronization between biological rhythms and social commitments and the consequent peak of performance in the afternoon, while no statistically significant difference between the two sessions was shown in HSJL.

Surprisingly, no significant difference between the two groups was found in YBT. These findings disagree with the current scientific literature reporting that postural control is altered with sleep deprivation (37, 38). In particular, sleep deprivation leads to deficits in various bodily functions (39), connected to disturbances in the circadian rhythms (17) which may affect neuronal and muscular functions, critical determinants of balance (40). We speculate that in our study there is no difference in balance skill between the two groups because generally volleyball players prioritize jump training over balance training, reducing the risk of deviation from reality in the former, while the latter is less considered and more susceptible to variations. Moreover, although not statistically significant, a slight difference between the two groups was observed. Perhaps, a larger sample size might have revealed stronger results in this test as well.

No significant results were found in the comparisons related to CT, probably due to the heterogeneity of the groups (only 3 M-types compared to 10 E- and 17 N-types).

### 4.1 Limitations of the study

This study has some limitations. Firstly, although field tests closely resemble the actual performance required by players, their accuracy and precision are often operator-dependent, which may have introduced errors in the measurements. Additionally, our sample is small and consists solely of young females, making it difficult to generalize the results to a broader population. Furthermore, SJL was assessed using a questionnaire; employing a more objective method might have yielded more reliable results.

## 5 Conclusion

It appears that the impact of SJL on exercise physiology is akin to that of sleep debt and training out of synchrony, affecting areas such as metabolism, temperature regulation, and psychological factors, which can potentially lead to decreased performance. Although the role of SJL in health is becoming more studied, few investigations have focused on its effects on physical performance. Our findings highlight how SJL can affect jump skills in volleyball players. Future research should explore its impact on other skills and sports, utilizing lab tests and objective evaluation methods. Additionally, studies investigating the physiological and biochemical pathways regulating this response may clarify the reasons behind these findings. In conclusion, coaches and trainers should recognize the importance of maintaining regular circadian rhythms and precise sleep schedules to optimize performance and reduce the risk of injury.

## Data Availability

The raw data supporting the conclusions of this article will be made available by the authors, without undue reservation.
